# Utility and Safety of Romiplostim in Pediatric Allogeneic Stem Cell Transplantation

**DOI:** 10.1111/petr.70228

**Published:** 2025-11-18

**Authors:** Srividhya Senthil, Abdul Moothedath, Jane Elizabeth Potter, Heather Mcgrath Wilkinson, Eden Whiteside, Ramya Nataraj, Omima Mustafa, Claire Horgan, Denise Bonney, Sarah Brett, Rob Wynn

**Affiliations:** ^1^ Department of Bone Marrow Transplantation Royal Manchester Children's Hospital Manchester UK; ^2^ Department of Pharmacy Royal Manchester Children's Hospital Manchester UK

## Abstract

**Background:**

The use of romiplostim, a thrombopoietin agonist, has increased in the last decade for the treatment of immune mediated thrombocytopenia and severe aplastic anemia. Its utility has been explored in the management of delayed platelet engraftment and secondary platelet failure during stem cell transplant (SCT), but its use has remained largely anecdotal in pediatric allogeneic SCT.

**Methods:**

In this single centre, retrospective study we report the largest pediatric SCT cohort use of romiplostim.

**Results:**

Romiplostim was used in 17 children for several indications, principally including poor graft function (PGF) and immune‐mediated cytopenia (IMC), including multi‐lineage cytopenia. The overall response rate (ORR) was 76.5% and the median time to achieve OR was 42 days. No toxicity was observed with romiplostim including marrow fibrosis, clonal evolution and thrombosis with a median follow‐up of 18 months. Romiplostim averted the need for second allogeneic SCT in two patients with late graft failure and the need for stem cell boost (SCB) in three patients.

**Conclusion:**

We propose that romiplostim can be safely used in the cytopenia in pediatric SCT to good effect.

**Abbreviations**
AML: acute myeloid leukemia
CLL: chronic lymphocytic leukemia
CMV: cytomegalovirus
CR: complete response
GF: graft failure
GVHD: graft‐versus‐host disease
HB: hemoglobin
HSV: herpes simplex virus
IMC: immune mediated cytopenia
IST: immunosuppressive treatment
ITP: immune mediated thrombocytopenia
JMML: juvenile myelomonocytic leukemia
OR: overall response
ORR: overall response rate
PGF: poor graft function
SCB: stem cell boost
SCT: stem cell transplant
STR: short tandem repeats
TMA: thrombotic microangiopathy
TPO: thrombopoietin
TPO‐RA: thrombopoietin‐ receptor agonist

## 1 Background

Romiplostim is a recombinant Fc peptide fusion protein which acts as a thrombopoietin agonist has been increasingly used in immune‐mediated thrombocytopenia (ITP) setting. More recently, it has been reported to have utility in severe aplastic anemia, and in delayed platelet engraftment and secondary platelet failure in post stem cell transplant (SCT). Conventional treatment of poor graft function (PGF) and immune‐mediated cytopenia (IMC) are CD34 selected stem‐cell‐boost (SCB) and immunosuppressive treatment (IST) respectively. We have previously reported a relationship between IMC and graft failure (GF), and that IMC might presage GF, a forme fruste [1]. There is a lack of data on the utility and safety of romiplostim in pediatric SCT. We report our significant experience of romiplostim in PGF and IMC in pediatric allogeneic SCT. We present our single‐centre experience of the use of romiplostim in a post stem cell transplant context in pediatric recipients in the last 5 years, which is to date, the largest cohort in pediatric allogeneic SCT setting. The indications were diverse, including PGF, delayed platelet engraftment and immune cytopenia. We also make a special note of its use in post cord blood transplant immune cytopenia and impending graft rejection, where it has successfully helped to salvage the graft, averting the need for a second stem cell transplant.

## 2 Methods and Materials

This is a retrospective study of pediatric allogeneic SCT recipients who received romiplostim for treatment of cytopenia between 2019 and 2025. The electronic case records were analyzed to get patient, donor and transplant characteristics, etiology of cytopenia and dose, duration, response and side effects to romiplostim. The primary etiology of cytopenia was classified into either IMC or PGF, taking into consideration, the cellularity of the bone marrow along with its morphology in conjunction with other tests such as direct antiglobulin test, anti‐platelet antibodies and anti‐neutrophil antibodies. The overall response (OR) is defined as achievement of platelet count > 100 × 10^9/L, absolute neutrophil count > 1 × 10^9/L and Hb > 80 g/L without transfusion support.

## 3 Results

Romiplostim was used in 17 pediatric patients in the post‐transplant setting. As indicated in Table 1, the cohort included mainly non‐malignant SCT patients with only four malignant SCT patients whose cytopenia was refractory to multiple lines of treatment. The median age of the cohort was 3 years (range: 1–16 years).

**TABLE 1:** Characteristics of Hematopoietic Stem Cell Transplant (HSCT) recipients where romiplostim was used in the study and the response variables.

Characteristics	Patient 1	Patient2	Patient 3	Patient 4	Patient 5	Patient 6	Patient 7	Patient 8	Patient 9	Patient 10	Patient 11	Patient 12	Patient 13	Patient 14	Patient 15	Patient 16	Patient 17
Age in years	1	1.5	2	5	1	3	7	1.5	2	10	3	16	11	2	16	4	2
Gender	F	F	M	M	M	F	M	F	M	M	M	M	M	F	M	m	m
Diagnosis	SAA	MPS‐1H	SAA	CHS	WAS	JMML	Beta Thalassemia	MPS1H	MPS 1H	Beta thalassemia	Relapsed AML	SAA	Therapy related AML	Monosomy 7 MDS	Sickle cell disease	MPS2	MPS1H
Donor type	MUD	MMUD	MUD	MMUD	MUD	MUD	MUD	MUD	MMUD	MSD	MUD	Haplo‐related	MMUD	MUD	Haplo‐related	MUD	MMUD
Stem Cell source	PBSC	CB	BM	CB	BM (cryopreserved)	CB	BM	BM	CB	BM	BM	BM and CD34 selected PBSC	CB	BM	BM and CD34 selected PBSC	CB	CB
TNC/kg of BW	11.4 × 10^8	15.4 × 10^7	0.98 × 10^8	0.738 × 10^7	8.8 × 10^8	2.4 × 10^7	5.4 × 10^8	6 × 10^8	8.16 × 10^7	4.8 × 10^8	6.48 × 108	1.85 × 10^8	3.65 × 10^7/kg	8 × 10^8	2.29 × 10^8	8 × 10^7	5.9 × 10^7
CD34/Kg of BW	10 × 10^6	15.36 × 10^5	0.75 × 10^6	1.6 × 10^5	6.06 × 10^6	1.5 × 10^5	4.3 × 10^6	5 × 10^6	3.18 × 10^5	4.5 × 10^6	4.2 × 10^6	4.25 × 10^6	1.02 × 10^5/kg	4.95 × 10^6	2.26 + 8.70 × 10^8	3.3 × 10^5	1.75 × 10^5
ABO mismatch	Nil	Major	Major	Minor	Nil	Nil	Minor	Minor	Major	Major	Nil	Minor	Nil	Nil	Nil	Nil	Nil
Conditioning	Flu/Cy	Flu/Bu	Flu/Cy	Flu/Treo	Bu/Flu	Flu/Bu	Flu/Treo/TT	Flu/Bu	Flu/Bu	Flu/Treo/TT	Bu/Cy	Flu/Cy	Flu/Treo/TT	Flu/Treo/TT	Flu/Cy	Flu/Bu	Flu/Bu
Recipient HLA antibodies	Nil	Nil	Nil	Nil	Nil	Nil	Nil	Nil	Nil	Nil	Nil	Nil	Nil	Nil	Nil	Nil	Nil
Neutrophil engraftment (days)	12	16	22	25	14	21	10	15	15	14	14	15	21	11	12	12	22
Platelet engraftment (days)	11	27	23	26	14	33	16	28	—	60	22	30	—	22	23	32	—
Chimerism on day 30 (%)	100	100	95	100	100	98.6	100	97	97.3	100	100	100	100	100	100	89.9	87.3
Chimerism on day 90 (%)	100	100	95	100	100	100	100	97.6	33.4	100	100	100	100	100	100	93.9	50
Last recorded whole blood (WB) chimerism (%), months post‐transplant when assessed.	100, 24	100, 18	42.2, 24	100, 24	100, 24	100, 24	100, 24	90, 24	n/a	100, 18	100, 6	100, 18	100, 9	100, 7	100, 6	96.4, 7	52.9, 9
Corresponding last recorded myeloid and lymphoid donor chimerism (%) when WB chimerism not fully donor.	n/a	n/a	34, 66	n/a	n/a	n/a	n/a	96.3, 82.5	n/a	n/a	n/a	n/a	n/a	n/a	n/a	100, 73.1	63, 14.2
Viral reactivation	Adenovirus	Nil	CMV	CMV	Adenovirus	Nil	Nil	Nil	CMV	CMV	Adenovirus, Primary EBV	EBV	Nil	Primary EBV	HSV, CMV	Nil	Nil
GVHD	No	Stage 3 skin	No	No	No	No	Stage 2 Skin	Stage 2 Skin	No	Stage 3 Skin	Stage 3 skin	Stage 2 Skin	Stage 1 Skin Stage 4 gut	Stage 1 skin	Liver	Stage 3 Skin	No
TA‐TMA	No	Yes	No	No	No	No	No	No	No	No	Yes	No	Yes	Yes	No	No	No
Cytopenia	Plt, Neut	Plt, Neut	Plt, Neut, Hb	Plt	Plt, Neut, Hb	Plt	Plt	Plt, Neut	Plt, Neut, Hb	Plt, Neut, Hb	Plt, Hb	Plt	Plt, Hb	Plt, Hb	Plt	Plt, Hb	Plt, Neut, Hb
Indication for Romiplostim	PGF	IMC	PGF	IMC	PGF	IMC	IMC	IMC	SGF	IMC	IMC	IMC	IMC	IMC‐ PGF	PGF	IMC	PGF
Cellularity in trephine (%)	30–40	80	50	10–20	5	80	20–30	80	10–20	85	80	40	60–70	50–60	20	80	10–20
Cause of Cytopenia	Adenovirus, Cidofovir	Alloreactivity, GVHD	Low stem cell dose, ABO mismatch, CMV, Ganciclovir	Alloreactivity, CMV, ganciclovir	Alloreactivity, Adenovirus, Cidofovir	Alloreactivity	Alloreactivity	Alloreactivity, Gram‐negative sepsis	Immune mediated rejection, CMV, Ganciclovir	Alloreactivity	Alloreactivity	Alloreactivity	Alloreactivity, TA‐TMA	Alloreactivity, TA‐TMA, HSV, Foscarnet	HSV, foscarnet, Flu	Alloreactivity	Immune mediated rejection
Post‐transplant day of initiating Romiplostim	105	66	72	195	327	812	373	240	42	85	39	163	167	167	55	43	63
Time to platelet response in days	120	43	180	35	90	12	28	13	Failed	78	Failed	34	Failed	Failed	45	9	42
Cumulative dose to platelet response (mcg/kg)	71	55	165	45	87	10	25	20	Failed	105	Failed	50	Failed	Failed	60	2	60
Time to neutrophil response in days	38	17	30	n/a	8	n/a	n/a	13	Failed	15	n/a	n/a	n/a	n/a	n/a	n/a	21
Cumulative dose to neutrophil response (mcg/kg)	17.5	15	35	n/a	5	n/a	n/a	20	Failed	15	n/a	n/a	n/a	n/a	n/a	n/a	30
Time to red cell response in days	n/a	n/a	28	n/a	105	n/a	n/a	n/a	Failed	15	Failed	n/a	Failed	Failed	n/a	8	28
Cumulative dose to red cell response (mcg/kg)	n/a	n/a	35	n/a	107	n/a	n/a	n/a	Failed	15	Failed	n/a	Failed	Failed	n/a	1	40
Time to overall response in days	120	43	180	35	90	12	28	13	Failed	78	Failed	34	Failed	Failed	45	9	42
Cumulative dose to overall response (mcg/kg)	71	55	165	45	87	10	25	20	Failed	105	Failed	50	Failed	Failed	60	2	60
Rebound thrombocytopenia	No	No	No	No	No	Yes	No	No	n/a	No	n/a	No	n/a	n/a	No	No	No
Adverse events	Nil	Nil	Nil	Nil	Nil	Nil	Nil	Nil	Nil	Nil	Nil	Nil	Discontinued due to pain associated with administration	Nil	Nil	Nil	Nil

Abbreviations: AML, acute myeloid leukemia; BM, bone marrow; BW, body weight; CB, cord blood; CHS, Chediak Higashi Syndrome; CMV, cytomegalovirus; EBV, Ebstein‐Barr virus; GVHD, graft‐versus‐host disease; HLA, human leucocyte antigen; HSV, herpes simplex virus; IMC, immune mediated cytopenia; JMML, juvenile myelomonocytic leukemia; MDS, myelodysplastic syndrome; MMUD, mismatched unrelated donor; MPS‐1H, mucopolysaccharidosis‐1 Hurler's syndrome; MPS‐2, mucopolysaccharidosis type 2; MUD, matched unrelated donor; n/a, not applicable; PBSC, peripheral blood stem cells; PGF, poor graft function; SAA, severe aplastic anemia; SGF, secondary graft failure; TA‐TMA, transplant associated thrombotic microangiopathy; TNC, total nucleated cell; WAS, Wiskott Aldrich syndrome.

The 17 cases included 4 cases of cytopenia related to PGF, 10 cases of IMC, 1 where cytopenia initially was due to IMC, progressed to PGF and 2 cases of secondary graft failure. ITP was the most common cytopenia in the IMC group (*n* = 4) followed by Evan's (*n* = 3) and the least common pattern was trilineage autoimmune cytopenia (*n* = 1). The commonest pattern of PGF was trilineage cytopenia (*n* = 2). The contributory factors implicated in cytopenia etiology, included alloreactivity where concurrent graft versus host disease (GVHD) of skin or gut was present (*n* = 11), adenovirus (*n* = 2), CMV (*n* = 3), HSV (*n* = 2), myelosuppressive drugs (*n* = 7), low stem cell dose (*n* = 1), ABO major incompatibility (*n* = 4), TMA (*n* = 2) and gram‐negative sepsis (*n* = 2).

Romiplostim was commenced, variably, from 39 to 812 days post‐transplant at a weekly dose of either 2.5, 5 or 10 mcg/kg depending on the depth of cytopenia and doses were escalated to a maximum of 10 mcg/kg to achieve the desired response. The patients earlier in the cohort were started at a lower dose of 2.5 mcg/kg/week and further doses were gradually escalated. However, most of the later patients were observed to have a starting dose of 10 mcg/kg/week.

**FIGURE 1:**
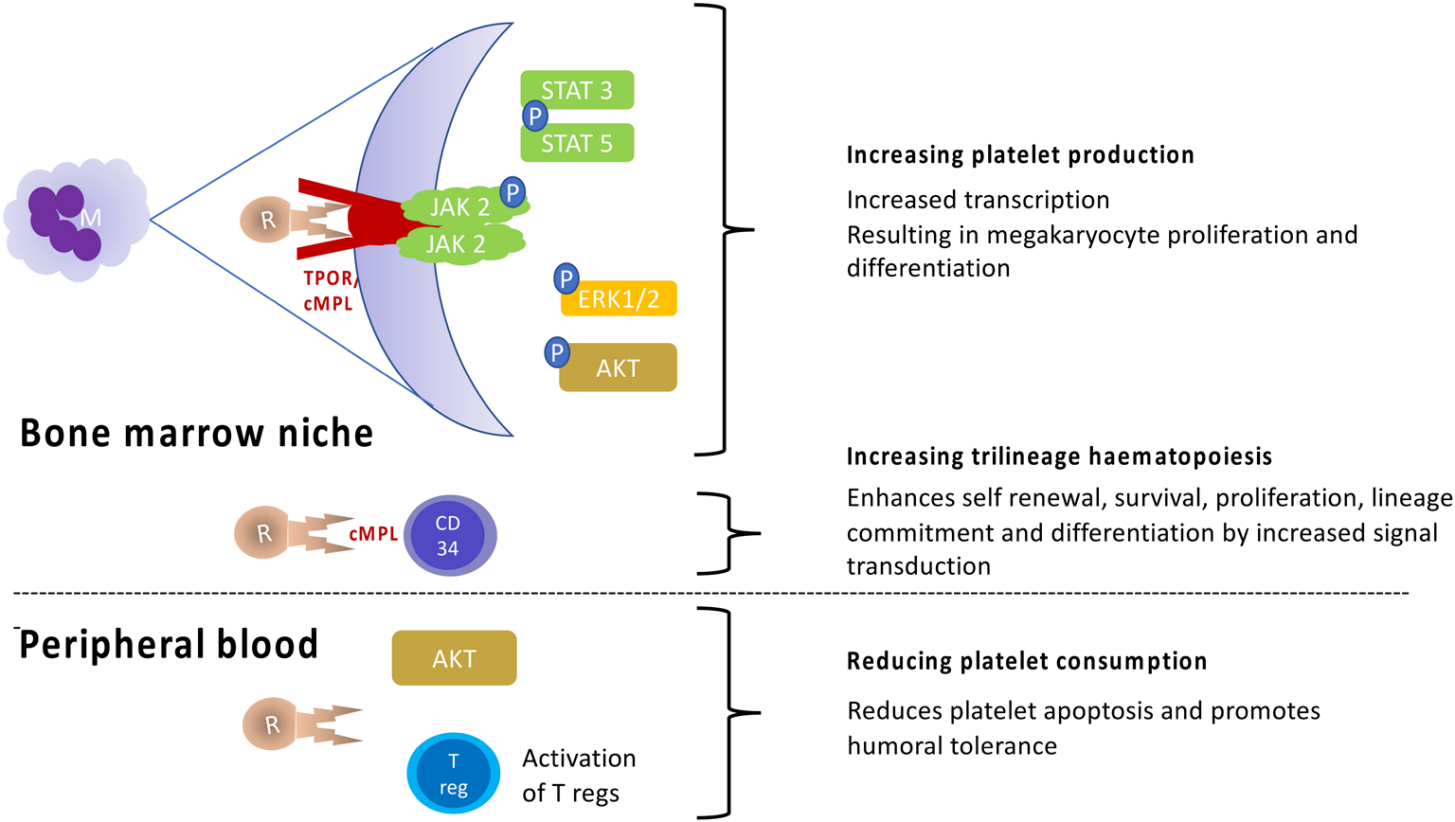
Cellular mechanism of action of romiplostim. Romiplostim binds to the c‐MPL receptor on the megakaryocyte, resulting in a conformational change that triggers activation of various downstream transcription signaling pathways including JAK–STAT, Akt, ERK pathways leading to megakaryocyte proliferation and increased production of platelets. Similar action on CD34 hematopoietic stem cells (HSC) results in improved trilineage hematopoiesis by a surge in self‐renewal, survival, proliferation, lineage commitment and differentiation of the HSCs. By its action on T regulatory cells via the Akt pathway, it improves humoral tolerance and reduces platelet apoptosis.

The overall response (OR) defined as achievement of platelet count > 100 × 10^9/L, absolute neutrophil count > 1 × 10^9/L and hemoglobin (Hb) > 80 g/L without transfusion support, was achieved in 13 of the 17 patients, giving an Overall Response Rate (ORR) of 76.5%. The median time to achieve OR was 42 days (range 9–180 days) and that to achieve platelet, neutrophil, red cell response was 42 (range:9–180), 17 (range: 8–38) and 28 (range: 8–105) days respectively. The median cumulative dose of romiplostim needed to achieve overall response was 55 mcg/kg. In all patients who had trilineage cytopenia, platelet response was the last to recover amongst all lineages. Failure of response was seen in four patients: one with secondary graft failure who underwent a second allogeneic SCT after two doses, one with fulminant, extensive autoimmunity refractory to many lines and in the other two where the cytopenia was multifactorial including alloreactivity, TMA and HSV infection. It is mentionable that of these four failed responses, two patients had only two doses of romiplostim before cessation Figure 1.

**FIGURE 2:**
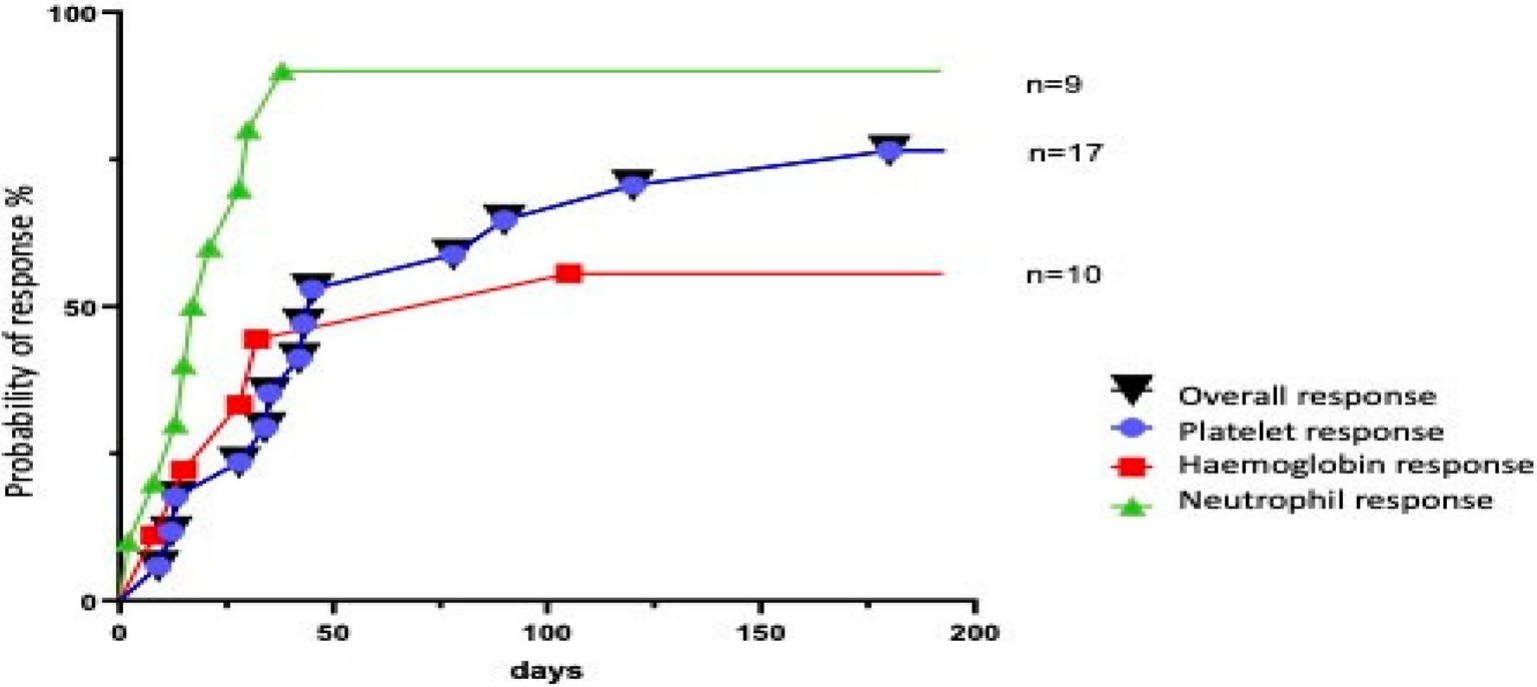
Plot of cumulative response to romiplostim versus time (in days). The black inverted triangle represents overall response (OR). The platelet, hemoglobin and neutrophil responses are depicted by a blue circle, a red square and a green triangle respectively. “*n*” indicates the total number of subjects within the study, in whom the particular cell lineage was affected.

The donor myeloid chimerism by short tandem repeat (STR) was maintained at 100% in all the patients except three, who had evidence of declining chimerism before commencement. While the graft function improved, and chimerism stabilized partially in two patients, the chimerism in the other patient continued to worsen, as detailed in Table 1. However, all three patients continue to maintain a normal full blood count with transfusion independence. The treatment response is maintained in all patients after discontinuation of the drug, without rebound cytopenia, except in one, where the drug was reintroduced to successfully treat rebound ITP, before the ultimate cessation of the drug. Though, platelet count was observed to fall slightly after cessation of the drug, in no other case did it progress to moderate or severe thrombocytopenia. The donor lymphoid chimerism, which carries little significance in SCT for non‐malignant indications, while it was variable, did not bear any correlation to the use of romiplostim in the study group Figure 2.

Importantly, Romiplostim averted the need for a planned second allogeneic SCT in two patients and planned SCB in three patients. It reduced the need for prolonged IST in eight patients in whom there were worrying concerns of concurrent serious infections.

No side effects noted at a median follow‐up of 18 months (range: 0–42 months). The patients who had PGF were found to have satisfactory trilineage hematopoiesis in their repeat marrow assessment after treatment with romiplostim, with no increase in reticulin deposition or clonal evolution. No thrombotic complications were observed in the cohort. Of the four malignant indications of stem cell transplant, where romiplostim was used, no disease relapse was observed within the follow‐up period. These four patients had regular bone marrow assessments up until 1 year post‐transplant and later, were closely monitored with routine chimerism and full blood count. Patient 13, in whom the drug was ceased after two doses, due to intolerance to subcutaneous injection, developed a donor‐derived myeloproliferative disorder and succumbed to disease‐associated morbidity.

## 4 Discussion

Thrombopoietin is a pivotal and potent glycoprotein hormone produced in the liver, kidneys and bone marrow which plays an important role in the production and maturation of megakaryocytes. It increases platelet production by its interaction through c‐MPL on megakaryocyte precursors and increases the number, size, ploidy and supports the formation and maturation of platelets through JAK2 and STAT5 pathways [2].

Apart from its action on megakaryopoiesis, it also supports the expansion and self‐renewal of HSCs and its action prevails on all progenitor cells which have the potential to differentiate to megakaryocyte [3]. In fact, it is crucial for the sustenance of viability of stem cells and maturation of early multilineage progenitor cells and erythroid progenitor cells. For this reason, children who are born without the TPO receptor as in congenital amegakaryocytic thrombocytopenia, though thrombocytopenic to begin with, ultimately become pancytopenic [4].

Thrombopoietin is produced at a steady rate from the liver, and it has an interesting negative feedback regulation. Thrombopoietin uptake is mediated through c‐MPL receptors on megakaryocytes and platelets [5]. While uptake in megakaryocytes leads to stimulation of thrombopoiesis, uptake in platelets leads to degradation of the hormone. Therefore, the higher the number of megakaryocytes and platelets, the lower is the level of thrombopoietin and vice versa. Exceptions are in ITP and ET, where TPO levels are disproportionately normal.

Romiplostim is a second‐generation TPO receptor agonist which is a recombinant 14 amino acid, Fc peptide fusion protein with four binding domains and competes with endogenous TPO for binding [6]. One of the binding domains, TPO‐R has a strong affinity to c‐MPL on the megakaryocytes. Unlike the first‐generation TPO agonists, romiplostim bears no structural homology to endogenous TPO; thereby, there is no issue of neutralizing antibodies developed against the endogenous TPO [7]. Romiplostim binds to the extracellular domain of TPO‐R on megakaryocytes and increases the tyrosine phosphorylation and thereby increases the transcription through JAK2–STAT5, ERK1/2, and AKT pathways [8]. Similar to other IgG molecules, it is protected from degradation by FcRn mediated uptake on endothelial cells and recycling, giving a half‐life of 120–140 h [9]. It acts on megakaryocyte CFUs and increases the number, size and ploidy of megakaryocytes. Romiplostim also acts through c‐MPL receptors on hematopoietic stem cells. Consequently, this property is being harnessed in treating trilineage cytopenia in severe aplastic anemia.

Thrombocytopenia can be caused either by decreased platelet production or by increased consumption. Romiplostim helps largely by increasing platelet production. Less well known is the fact, that romiplostim has an immunomodulatory property, by which it tackles the problem of platelet destruction. It reduces platelet apoptosis by signaling through the AKT pathway [10]. The Fc region of romiplostim confers some immunomodulatory properties including increasing the activation of T regs and promoting humoral tolerance through interaction with Fc gamma receptors [11].

Thrombocytopenia post stem cell transplant is observed in around 5%–20% of the cases which increases the morbidity and mortality associated with the transplant [12]. It can either be due to primary delayed platelet engraftment or secondary failure of platelet response or associated with poor graft function (PGF).

Poor graft function (PGF) is defined as the cytopenia in at least two cell lineages post stem cell transplant associated with a hypocellular marrow with full donor chimerism in the absence of GVHD or other reversible causes [13]. It is usually caused by low stem cell dose, irreversible damage done by viruses and myelotoxic drugs.

The failure of hematopoietic recovery by Day 30 following an allogeneic transplant with poor or no detectable donor chimerism is called primary graft failure. Decline in hematopoietic function necessitating blood transfusion support and/or growth factor support, associated with falling donor chimerism is termed as secondary graft failure. Graft failure is caused by an immune‐mediated rejection of donor cells by the host immune system.

To date, at least 30 retrospective studies reporting the use of TPO agonists are documented. There are at least six ongoing clinical trials, testing the use of TPO‐RA in the realm of post‐transplant thrombocytopenia and poor graft function [14].

De Latour et al. outline the results of a phase I/II multicentre prospective trial of using romiplostim in adult allogeneic transplant patients with either delayed platelet engraftment or secondary transfusion‐dependent thrombocytopenia [2]. Patients were eligible if the platelet count remained less than 20 for 7 days after day 45 of the transplant or remained or became transfusion dependent. Of the 19 patients who received all 12 doses of romiplostim, 18 responded with a platelet count of > 50. A total of 17 had durable responses and the median time to achieve a platelet count > 50 was 45 days. There was no increase in relapse or non‐relapse mortality in those cases. There was no increase in marrow reticulin in 6 weeks and 1 year follow‐up marrow.

Scordo et al. reported the results of a single‐arm pilot study using romiplostim from D + 1 in autologous stem cell transplant in adult recipients [15]. Romiplostim did not decrease the duration or the depth of thrombocytopenia post‐transplant. However, it augmented the platelet recovery after D + 15.

Christakapoulos et al. give an account of the utility of weekly romiplostim in accelerating platelet engraftment in cord blood transplant recipients from a phase I study [16]. There was accelerated engraftment of platelets both to levels of 20 and 50.

Kantarjian HM et al. conducted a 5‐year prospective randomized controlled trial in low‐risk MDS patients treated with romiplostim and a placebo [17]. Romiplostim significantly decreased the number of platelet transfusions and clinically significant bleeding events compared to the placebo. However, the study was halted due to concerns of an increase in blast percentage and potential risk of progression to AML, though the 5‐year overall survival and AML incidences were similar across both groups in the end.

Lancman et al. describe a small case series, where romiplostim was used in primary delayed platelet engraftment and in secondary failure of platelet response in five adult patients who received allogeneic stem cell transplant including for malignant and non‐malignant indications [18]. Of the five patients, four patients had a response and the response was sustained in the absence of relapse.

LM Poon et al. describes the utility of romiplostim in three adult patients with primary delayed platelet engraftment or secondary platelet response failure following allogeneic stem cell transplant, one of whom might have had an immune‐related platelet destruction while others were primarily due to PGF [19].

Al Mashdali et al. report the successful use of romiplostim in four adult patients who had thrombocytopenia either due to primary or secondary poor graft function [20].

The above quoted evidence is based on adult allogeneic bone marrow transplants and there is scarce data to date, on romiplostim use in pediatric bone marrow transplant recipients. Maximova et al. describe the efficacy of the drug in achieving platelet transfusion independence in six out of the seven patients with secondary failure of platelet recovery post allogeneic SCT [21].

The time‐tested definitive treatment of poor graft function is CD34 selected stem cell boost, which does not preclude the potential risk of complications including GVHD and inaccessibility. Shahzad et al. reported a systematic review on CD34 selected stem cell boost for poor graft function in allogeneic stem cell transplant recipients [22]. The CR and ORR in 209 patients analyzed were 72% and 80% respectively. Despite selecting CD34 stem cells, which helps to reduce the potential of GVHD, the risk is not completely alleviated and the incidence of acute and chronic GVHD after SCB was found to be 17% and 18% respectively. Another retrospective study also demonstrates a similar ORR in a group of 41 patients treated with CD34 selected SCB with comparable acute GVHD rates [23].

Most of the studies on romiplostim suggest a similar, if not better outcome in the correction of cytopenia, avoiding the unwanted side effect of GVHD. Moreover, in cases of cord blood transplant where a stem cell boost is not feasible, romiplostim appears to be a beneficial strategy to avoid a second transplant for the correction of poor graft function.

In our cohort, romiplostim averted the need for a second transplant in two patients with poor graft function—one who was a cord blood transplant recipient for Hurler's syndrome and the other being severe aplastic anemia who had falling donor chimerism following a matched unrelated donor transplant. In both instances, the CD34 SCB would not have been feasible. Besides, it helped obviate the need for stem cell planned in three patients (Patient 1, Patient 5, Patient 15).

Immune mediated cytopenia incidence post SCT has been variably reported to occur in up to 22% of the cases [24]. Conventionally, the treatment of IMC has been intensifying immune suppression post‐transplant with a variety of treatment agents including steroids, MMF, rituximab, bortezomib and the like. The treatment efficacy is variable. These treatment agents do bring in the complications of infections including viral reactivations and fungal infections. Owing to the associated immunomodulatory property, we believe, romiplostim can be used to treat these IMC, without enhancing the risk of infection. Previous studies have been instructive that IMC can be a harbinger of graft rejection, particularly, in cord blood transplantation [1]. We would like to specifically highlight the successful salvage of the graft from an immune mediated graft failure following a cord blood transplant in Hurler's syndrome (Patient 17). While it is an encouraging observation, we appreciate that it is an isolated case and its utility in such situations needs to be tested in a larger cohort.

The median effective dose was found to be 4–5 mcg/kg/week [12]. While many studies and guidelines suggest starting romiplostim at a dose of 1 mcg/kg/week and increasing the dose weekly by 1mcg to achieve the desired platelet count of 50–250 [12, 13], some suggest starting at higher doses of 3 mcg/kg/week or even at 10 mcg/kg where an urgent response is wanted and to know if the drug is going to be effective within a short timeframe. Some studies suggest withholding the dose in case of a platelet count higher than 400 and few warn regarding a potential sharp fall in the count with that strategy [25] and recommend a weaning dose by 50% in such instances, instead. In our cohort, we have commenced the drug at various doses ranging from 2.5 to 10 mcg/kg/week. Nevertheless, we believe that starting the drug at 10 mcg/kg/week helped achieve quicker responses, without producing any significant side effects. We weaned the drug after correcting the cytopenia by 2.5 mcg/week every 2 weeks before complete cessation, with which we did not notice any significant rebound cytopenia.

The most frequent side effects experienced by adults on romiplostim were fatigue and headache. There were no clinically significant anti‐TPO neutralizing antibodies developed during romiplostim treatment [26]. Though two patients developed hematological malignancies (CLL, lymphoma) whilst on treatment with romiplostim, both were found to have evidence of disease prior to the start of the treatment. The consensus of all studies that investigated marrow reticulin post‐romiplostim treatment was that there is an increased reticulin in some patients on romiplostim treatment for ITP. However, the increase in reticulin was infrequently significant, the highest being reported was grade 3 and did not appear to have any clinical effects. This marrow reticulin was reversible with cessation of treatment with romiplostim [27, 28].

We did not notice any side effects in our treatment cohort. The patients who had poor graft function treated with romiplostim, had marrow assessments after completion of treatment which did not reveal any increase in reticulin deposition or clonal changes. However, we realize that the follow‐up of this cohort is short and long‐term follow‐up is needed to reassuringly exclude the risk of clonal transformation. Our cohort included mainly non‐malignant patients with the exception of four patients who had transplant for malignant indications—one was JMML with ITP who was in remission 2 years post‐transplant and the three others being AML post‐transplant in remission with refractory consumptive cytopenia. There is quite an understandable concern and reluctance to use an early‐acting cytokine on stem cells, in a post‐transplant setting for malignant indications for the fear of increasing the relapse risk. Though Patient 13, developed donor‐derived leukemia after a cord blood transplant, we believe that romiplostim had no causative role, as only two doses of the drug were administered in total. Long‐term follow‐up studies are needed to find out the actual relapse risk in such a population with romiplostim treatment, before freely recommending its use in those indications.

The limitations of this study include its retrospective nature, relatively small cohort and the lack of randomization between romiplostim and the standard alternatives as well as a shorter follow‐up period to reassure safety. While we acknowledge these limitations, we are strongly encouraged by the outcomes witnessed, in comparison to the historical outcomes achieved with standard alternative lines of management, balancing the associated side effects and the cost involved. Randomized studies with long‐term follow‐up are imperative and crucial to conclude the efficacy and safety of the drug.

## 5 Conclusion

This is the largest pediatric SCT‐based study on romiplostim to date, highlighting not only its role in PGF but also in IMC and GF. Romiplostim is a safe, effective and cost‐efficient alternative to SCB, second allogeneic transplants, prolonged IST and its ensuing complications in these settings. We recommend its promotion over other toxic approaches in these settings and a larger survey of its use and prospective studies with longer follow‐up.

## Author Contributions

R.W. proposed the study design. A.M., H.M.W., E.W. collected the necessary retrospective data for the study. S.S. drafted and R.W. edited the manuscript. All other co‐authors have reviewed the content in the manuscript. It is a non‐funded study.

## Conflicts of Interest

The authors declare no conflicts of interest.

## Data Availability

The data that support the findings of this study are available from the corresponding author upon reasonable request.
